# High-pressure intrapleural chemotherapy: feasibility in the pig model

**DOI:** 10.1186/1477-7819-10-29

**Published:** 2012-02-06

**Authors:** Olivier Facy, Pierre-Benoit Pages, Pablo Ortega-Deballon, Guy Magnin, Sylvain Ladoire, Bernard Royer, Bruno Chauffert, Alain Bernard

**Affiliations:** 1 INSERM Unit 866, Equipe Avenir, Dijon, France; 2 Department of Digestive Surgical Oncology, University Hospital, Dijon, France; 3 Department of Thoracic Surgery, University Hospital, Dijon, France; 4 Department of Anaesthesiology, University Hospital, Dijon, France; 5 GF Leclerc Regional Anticancer Center, Dijon, France; 6 Laboratory of Clinical Pharmacology, University Hospital, Besançon, France; 7 INSERM, UMR645, Besançon, France

**Keywords:** intrapleural intracavitary chemotherapy, pressure, pig model

## Abstract

**Background:**

The usual treatments for pleural malignancies are mostly palliative. In contrast, peritoneal malignancies are often treated with a curative intent by cytoreductive surgery and intraperitoneal chemotherapy. As pressure has been shown to increase antitumor efficacy, we applied the concept of high-pressure intracavitary chemotherapy to the pleural space in a swine model.

**Methods:**

Cisplatin and gemcitabine were selected because of their antineoplasic efficacy in vitro in a wide spectrum of cancer cell lines. The pleural cavity of 21 pigs was filled with saline solution; haemodynamic and respiratory parameters were monitored. The pressure was increased to 15-25 cm H_2_O. This treatment was associated with pneumonectomy in 6 pigs. Five pigs were treated with chemotherapy under pressure.

**Results:**

The combination of gemcitabine (100 mg/l) and cisplatin (30 mg/l) was highly cytotoxic in vitro. The maximum tolerated pressure was 20 cm H_2_0, due to haemodynamic failure. Pneumonectomy was not tolerated, either before or after pleural infusion. Five pigs survived intrapleural chemotherapy associating gemcitabine and cisplatin with 20 cm H_2_O pressure for 60 min.

**Conclusions:**

High-pressure intrapleural chemotherapy is feasible in pigs. Further experiments will establish the pharmacokinetics and determine whether the benefit already shown in the peritoneum is also obtained in the pleura.

## Background

Malignant pleural effusion is a common evolution of many cancers, including breast, lung or ovarian cancer. Today pleural mesothelioma is more frequent due to occupational exposure to asbestos [1]. Whereas peritoneal carcinomatosis may be treated by cytoreductive surgery followed by intraperitoneal chemotherapy with curative intent, the treatment of malignant pleural effusion is generally purely palliative [2,3]. Chemical or physical pleural abrasion, pleurodesis with sclerosing agents or talc are palliative treatments usually administered with concomitant systemic chemotherapy. Their objective is to alleviate the dyspnea but not to sterilize the pleural tumor [4]. As the pleura has the same histology as the peritoneum, we hypothesized that isolated pleural carcinomatosis could sometimes be treated with curative intent, as for the peritoneum, through the surgical resection of macroscopic malignant tissue associated with optimal intrapleural chemotherapy. Furthermore, this treatment may enhance local control of malignant pleural effusion. The major mechanism of failure for intracavitary chemotherapy is poor drug penetration into the depth of the tumor nodules and contaminated serous tissue [5]. We recently proposed increasing infusion pressure in the abdomen in order to enhance diffusion of the anticancer drug into the peritoneum [6]. The aim of the present study was to assess the set-up and the feasibility of high-pressure intrapleural chemotherapy for the treatment of pleural malignancies using a pig model. Due to their wide antitumor spectrum demonstrated by in vitro tests, we selected gemcitabine and cisplatin, and evaluated tolerance to this combination of drugs when used for high-pressure intrapleural chemotherapy.

## Methods

### In vitro cytotoxicity assay

The antitumor activity of cisplatin and gemcitabine against human breast (T47, MCF7), lung (H348, H1650), colon (SW480, HCT116) and ovarian (OVCAR3, IGROV1) cancer cell lines was evaluated according to a quantitative clonogenic assay in order to choose the drug and the doses for further in vivo experiments [7]. The target was to kill at least 80% of these human cancer cells after a 60 min of in vitro treatment. Cells were obtained from ATCC (Rockville, MD) except for IGROV1 cells, which were a gift from Dr J. Benard [8]. Briefly, cancer cells were seeded at low density (50 × 10^4^/well) in 24-well tissue culture plates for 48 hours before 60 minutes of exposure to the drugs. The cells were cultivated again in a drug-free culture medium for 7 days. Surviving adherent cells were fixed with pure ethanol and stained with 1% crystal violet. After washing with water and dye elution with 33% acetic acid, the optical density (OD) was read at 550 nm on a spectrophotometer. Survival was calculated as the OD in treated wells compared with that in untreated control wells.

### Animals

Before the experiment, 21 3-month-old large white female pigs, weighing 50-60 kg were allowed to acclimatize to the laboratory environment for 7 days with free access to standard food and water. The Animal Ethics Committee of the University of Burgundy, France approved the experiments (A0809).

### Anesthesia

Anesthesia was induced by intramuscular injection of 1 g ketamine + 1 mg atropine then completed with intravenous ketamine and sufentanil until the endotracheal intubation, without selective bronchic intubation. The animals were maintained under anesthesia by isoflurane (1.5%), intravenous sufentanil and cisatracurium. The tidal volume was maintained between 500 and 600 ml with a frequency of 25/min, adapted to the oxygen blood saturation. The bladder was catheterized to measure urine output. Systolic systemic arterial blood pressure (SAP) was monitored through a catheter inserted into the brachial artery. The heart rate (HR), electrocardiogram, nasal temperature, oxygen blood saturation (OS), cardiac output (CO in l/min) and cardiac index (CI in l/min/m2) were measured using the NICO system (Novametrix Medical Systems Inc., Wallingford, CT). Antibioprophylaxis with 2 g. of amoxicilline + clavulanic acid was given before the incision. Fluid resuscitation was achieved with isotonic saline, Ringer lactate and gelatine solution (Plasmion, Fresenius Kabi France, Sèvres, France) according to cardiologic ultrasonography parameters. No vasoactive drugs were used. Tramadol and paracetamol were prescribed for postoperative analgesia.

### Surgery

The anesthetized pigs were placed in the supine position. Two pleural catheters (Pleurocath^®^, Prodimed, Plessis-Bouchard, France), one for infusion and one for pressure recording, were inserted into the pleural cavity through the 5^th ^intercostal space. Saline solution was perfused by gravity in increasing steps until haemodynamic or respiratory intolerance was achieved. Six pigs underwent right pneumonectomy either before or after the pleural infusion. Right thoracotomy was performed in the 5^th ^intercostal space with the animals lying in left lateral decubitus. The accessory bronchial branch to the right superior lobe, always present in pigs, was ligated. The right bronchial branch, the pulmonary vein and artery were then ligated and pneumonectomy was performed including the accessory inferior lobe. The thoracic wound was closed on a pleural catheter if post-operative infusion was scheduled. Surviving animals had a daily visit and were kept in licensed animal facilities for 30 postoperative days until they were killed with Dolethal^® ^(Vetoquinol, France) and autopsied. Biological parameters (blood cell count, and serum levels of sodium, potassium and creatinine) were recorded before and after the surgery, and then on postoperative days 3, 7, 14 and 28.

### Anticancer drugs

Cisplatin (Sigma-Aldrich, L'Isle d'Abeau, France) and gemcitabine (Lilly, Suresnes, France) were diluted in isotonic saline solution (9 g/l NaCl) at 100 mg/l and 30 mg/l respectively and warmed to 37°C.

### Drug assay

Systemic blood and pleural liquid were sampled in five pigs at 0, 10, 20, 30, 40, 50 and 60 minutes after the beginning of the procedure. The samples were centrifuged and kept frozen at -20°C until the assays. The platinum concentration was measured by atomic absorption spectrometry (AAS) using a Zeeman atomic absorption spectrometer (Spectra-A; Varian, Les Ulis, France). Gemcitabine and 2,2-difluoro-2-deoxyuridine (dFdU) were assayed following the method described by Bowen et al [9]. Briefly, the serum was separated from whole blood samples and underwent protein precipitation by methanol after the addition of internal standards (13C and 15N isotopic gemcitabine and dFdU). Pleural liquid samples were simply diluted in distilled water. These serum and pleural samples were then derivatized with dansyl chloride, and dansyl-derivatives were extracted with methyl tertiary-butyl ether, evaporated and dissolved in a mixture of acetonitril and water (25/75). The samples were then assayed using high performance liquid chromatography (HPLC) coupled with Thermo Scientific tandem mass spectrometry. Chromatographic separation was performed using an Atlantis^® ^column (3 μm, 100 × 2.1 mm - Waters, USA) and the transitions used were 497.0 → 111.9, 500.0 → 114.9, 498.1 → 236.9 and 501.0 → 115.8 for the dansyl-derivatives of gemcitabine, isotopic gemcitabine, dFdU and isotopic dFdU, respectively. This method yielded a quantification limit of 0.010 μg/ml and was linear over the range of the concentrations studied.

## Results

### Tolerance to intrapleural pressure

Two pigs (**animals 1 and 2**) underwent percutaneous pleural infusion into the left thorax. At a pressure of about 15 cm H_2_O (11.5 mmHg), the mediastinal pleura broke in both pigs and the filled right pleural cavity was confirmed by ultrasonography and autopsy. Intrapleural pressure could not be maintained, haemodynamic parameters (systemic arterial pressure, cardiac output) failed and the animals died. Due to this fragility of the mediastinal pleura in the pig, further experiments were always performed on the right pleura (table 1).

**Table 1:** Synthesis of methods and results of all animals treated.

Pig	Side	Pneumonectomy	Chemotherapy	Pressure (cmH_2_0)/time (min)	Outcomes
1 2	Left	No	No	15/5	Left to right pleural filling

3 4	Right	No	No	15/30	Well tolerated

5 6	Right	No	No	20/30	Moderate haemodynamic changes
	
7	Right	No	No	20/60	

8	Right	No	No	25/5	Major but reversible haemodynamic instability

9 10	Right	No	No	25/5	Peroperative death

11	Right			15/15	Controlateral breach
				
12		Before infusion	No	20/5	Peroperative death
				
13				15/15	Major but reversible haemodynamic instability Postoperative death

14 15 16	Right	After infusion	No	20/40	Moderate haemodynamic changes Postoperative death

17 18 19 20 21	Right	No	Cisplatin (30 mg/l) Gemcitabin (100 mg/l)	20/60	Moderate haemodynamic changes No morbidity Pleurodesis

Moderate haemodynamic changes are defined as a decrease in arterial pressure and cardiac output requiring vascular resuscitation. When haemodynamic modifications persisted, they were major, and reversible if emptying saved the animal, or lethal if not.

Two pigs underwent a percutaneous procedure in the right thorax (**animals 3 and 4**). A pressure of 15 cm H_2_O was well tolerated for 30 minutes, with slight haemodynamic modifications (SAP showed a 15% decrease, HR increased by 25%, OS and CO decreased by 10%). A sustained pressure of 20 cm H_2_O (15.4 mmHg) on the pleura was obtained for 30 min or 60 min in 3 other animals (**5, 6 and 7**). This pressure was obtained in 1 hour by pleural filling with 4 l of isotonic saline solution. However, the fall in the systemic arterial pressure and cardiac output made it necessary to slow down the pleural inflow, mainly when a pleural pressure of 15 to 20 cm H_2_O was reached. The slow infusion of a mean of 500 ml of additional saline was required to maintain pleural pressure at 20 cm H_2_O. At this time of the procedure, the peak inspiratory pressure reached 45 cm H_2_O. Increasing pleural pressure to 25 cm H_2_O (20 mmHg) was tested in three animals (**8, 9 and 10**), but resulted in a decrease in systemic arterial pressure to below 40 mm Hg, and in the cardiac index to below 1.5 l/m^2^/min (table 2). Haemodynamic instability was associated with ECG signs of ischemia. In one animal, haemodynamic failure was reversible after emptying the pleural cavity but two other pigs died despite emptying, fluid resuscitation and cardiac massage.

**Table 2:** Mean and standard deviations of haemodynamic and respiratory parameters before (0 mmHg) and during infusion (15, 20 and 25 mmHg).

Intrapleural pressure	Heart Rate (/min)	Systolic Systemic Arterial Pressure (mmHg)	Cardiac Output (l/min)	Oxygen Saturation (%)	Ventilation pressure (cm H_2_O)
0 cm H_2_O	99.2 +/- 10.9	69.7 +/- 10.9	7.1 +/- 1	98.9 +/- 0.9	27.7 +/- 4.1

15 cm H_2_O	124.5 +/- 5.6	59.8 +/- 9.3	6.3 +/- 0.9	90.1 +/- 0.7	35 +/- 3.2

20 cm H_2_O	140.3 +/- 8.9	48.9 +/- 8.5	4.8 +/- 0.7	90.3 +/- 1	45.2 +/- 2.8

25 cm H_2_O	158 +/- 7.3	40.2 +/- 4.5	2.8 +/- 0.6	86.2 +/- 2.3	49.8 +/- 3.8

Pleural pressure was then combined with pneumonectomy corresponding to the treatment of mesothelioma. Three pigs had lung resection followed by pleural infusion (**11, 12 and 13**). Severe haemodynamic, electric and ultrasonographic alterations occurred with 15 cm H_2_O pleural pressure. The first pig presented right to left filling and died. The second pig also died when we attempted to increase the pressure to 20 cm H_2_0. The third animal survived the operation (but the infusion had to be shortened to 15 minutes at a pressure of 15 cm H_2_O), but died on the first post-operative day. Three animals had the reverse sequence, with the pleural infusion first, and then the right lung resection (**14, 15 and 16**). An intrapleural pressure of 20 cm H_2_O was easily tolerated for 30 minutes after filling with a mean of 4 l of saline solution infused in dorsal decubitus. After emptying the pleura, the animals were placed on their left sides and pneumonectomy was performed. Despite immediate survival, the 3 animals died on the first post-operative day.

### Selection of an active combination of drugs

**Figure 1:**
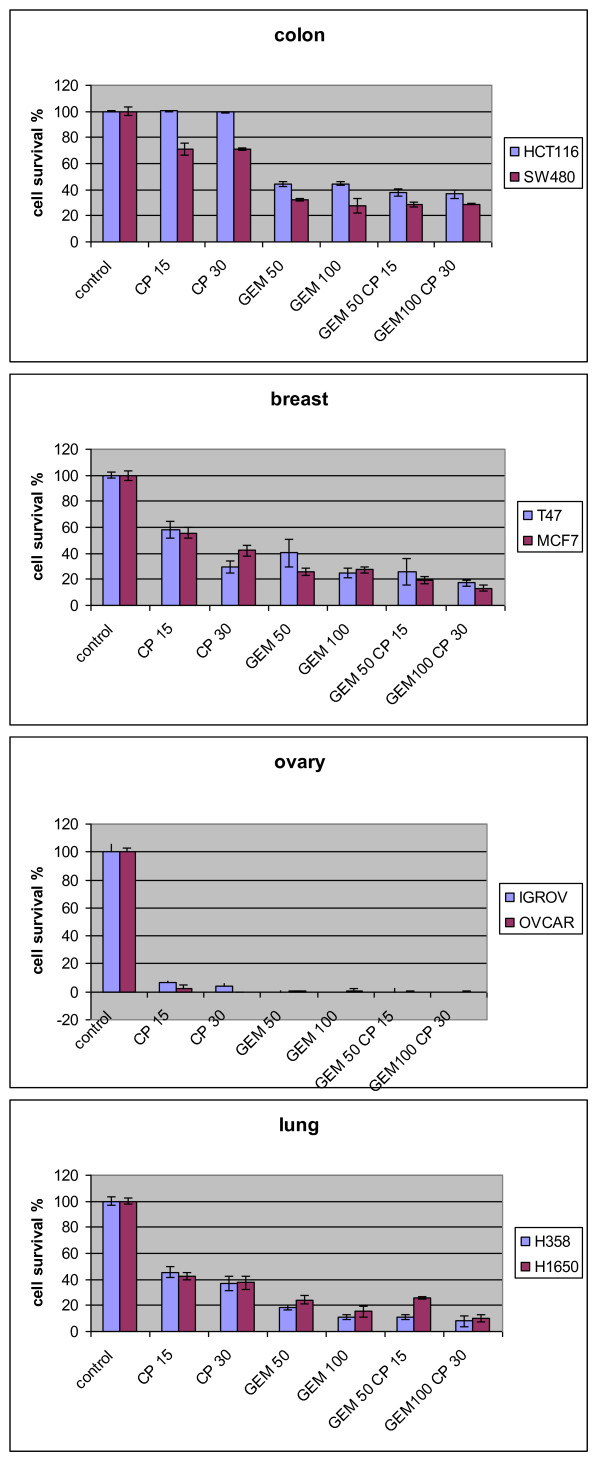
***In vitro *assay of 15 mg/l (CP 15) and 30 mg/l cisplatin (CP 30) and 50 mg/l (GEM 50) and 100 mg/l gemcitabine (GEM 100) on human colon, breast, lung and ovarian cancer cells**. Cell survival was analyzed 7 days after 60 minutes of exposure to the drug or their association.

Gemcitabine and cisplatin were tested alone or in combination with the target to kill at least 80% of various human cancer cells after 60 minutes of in vitro treatment (Figure 1). The objective was achieved with a combination of 100 mg/l gemcitabine and 30 mg/ml cisplatine for lung (H458, H1650) and breast (T47, MCF7) cancer cells but not for colon cancer cells (SW480, HCT116). Both ovarian cancer cell lines (OVCAR3 and IGROV1) were extremely sensitive to both drugs.

### Tolerance to intrapleural chemotherapy with pressure

Intrapleural hyperpressure chemotherapy combining 30 mg/l cisplatin and 100 mg/l gemcitabine for 60 min was finally performed in 5 animals (**17, 18, 19, 20 and 21**). The intrapleural chemotherapy was well tolerated. A pressure of 20 cm H_2_O was maintained for 40 (animal 17) or 60 min (animals 18, 19, 20 and 21). All of the animals survived with a normal clinical status, as well as normal blood cell count, creatinine and ionogram during the first month. At autopsy, tight pleurodesis was observed, and pathological examination of pulmonary samples showed a normal parenchyma. Pharmacokinetic analysis showed a stable pleural concentration of cisplatin throughout the infusion with a low systemic concentration. In the same way, constant high levels of gemcitabine were achieved in the pleural cavity. Almost no gemcitabine was metabolized to dFdU. Systemic levels of both drugs were very low, less than 0.1% of the pleural infusion.

## Discussion

Intrapleural chemotherapy has been used for a long time to treat malignant pleural effusion, but its efficacy is poor [10-15]. Some physical parameters, like hyperthermia, which is usually associated with intraperitoneal chemotherapy, are used to enhance drug uptake in cancer cells and cytotoxicity [13,14]. We previously demonstrated that increasing the abdominal pressure by filling the closed abdominal cavity resulted in the increased penetration of cisplatin into peritoneal tumor nodules and enhanced its antitumor activity in a murine model [6,16]. One of the known limits of anticancer drugs in intracavitary treatment is their poor penetration into tissues [5,17], due to the peritoneal barrier, which is histologically the same as the pleural barrier [18,19]. Extrinsic pressure could counterbalance the interstitial pressure, which is high in solid tumors (18 cm H_2_O in breast cancer, 27 cm H_2_O in cervical cancer, 23 to 41 cm H_2_O for head and neck cancer, 47 cm H_2_O in renal cancer, and 7 to 37 cm H_2_O in melanoma xenografts) [20]. Moreover, increased pressure in the cavities counteracts the hydraulic capillary pressure, which limits the diffusion of anticancer drugs from the cavity into tumor nodules. A pressure of 50 cm H_2_O (40 mm Hg) can easily be achieved for 2 hr in the abdomen of ventilated pigs after an average infusion of 8 l saline solution [6]. In non-ventilated rats, an abdominal pressure of only 26 cm H_2_O (22 mm Hg) was achievable after infusion of an average of 120 ml in 300-350 g animals. Nevertheless, this low level of pressure resulted in a 3-fold increase in platinum concentration in the tumour nodules compared with the same administration without hyperpressure. As a consequence, rats bearing millimetric peritoneal carcinomatosis were cured after intraperitoneal cispatin chemotherapy with pressure whereas rats which received conventional intraperitoneal injections were not [6]. This prompted us to assess the feasibility of pleural infusion with hyperpressure.

Intrapleural pressure is more difficult to achieve than abdominal pressure in pigs. In the present study, a pressure of 25 cm H_2_0 could not be reached in the right pleura of ventilated pigs. A lower level (20 cm H20) was well tolerated for up to 1 hr. The inspiratory plateau pressure, necessarily lower than 35 cm H2O, was not measurable in our ventilation machine [21]. Nevertheless, the clinical tolerance was good despite a high peak inspiratory pressure. Performing lung biopsies at the end of the procedure and monitoring of ventilatory parameters (like plateau pressure) may increase the safety of this approach. A pressure of 20 cm H_2_O was sufficient to at least double the concentration of platinum in peritoneal tumour nodules in rats (unpublished data). Pleural filling had to be slow (average 1 hour for 4 l). Monitoring of the pleural pressure made it possible to adapt filling to haemodynamic changes which appeared above 15 cm H_2_0. Vascular filling was modified according to cardiac ultrasonography monitoring. In the case of major haemodynamic failure, quick pleural emptying made it possible to reverse the situation. Factors which limit pleural pressure appear to be related more to mediastinal compression (heart, aorta, vena cava) than to the controlateral lung compression. Poor tolerance to increased pressure in the pleural compartment is well known, whatever the cause (tension pneumothorax or liquid effusion). This is more related to haemodynamics than to oxygenation disturbances [22,23]. In our experience, the first two parameters that dropped were cardiac output and systemic arterial pressure, before the fall in blood oxygen saturation. We think that the best ways to enhance tolerance to this procedure are slow infusion and efficient vascular filling. These appear better than decreasing tidal volume and increasing ventilation frequency.

Despite the well-known similarities to humans in terms of anatomy and physiology, the pig model has some drawbacks. Like humans, pigs have two separate pleural cavities, while rats have a single pleural space. However the mediastinal separation is thin and fragile in the pig. As a consequence, filling the left pleura twice resulted in fluid leakage to the right through a mediastinal breach, which occurred at a pressure of only 15 cm H_2_O. In contrast, filling the right pleura did not result in pleural breach. This is probably due to a protective effect of the median inferior lobe in the right pleural space. In humans, the separation between the right and left pleural compartments is thicker and the risk of leakage is probably similar after left or right infusion, given that any difference between the left or right pleura is not typical in clinical conditions, like hypertensive pneumothorax [23]. The second drawback is the poor tolerance of pigs to pneumonectomy, which is consistent with the findings of other authors [24]. Moreover the thin mediastinal pleura must be carefully protected during right lung resection because of the risk of injury and leakage. In addition, haemodynamic tolerance to pleural filling was poor (15 cm H_2_O was barely tolerated) when previous pneumonectomy had been performed. In contrast, initial pleural filling followed by pneumonectomy was tolerated just as well as in animals without lung resection. This is a major limit of the pig model for the preclinical evaluation of high-pressure intrapleural chemotherapy and lung resection as a treatment for mesothelioma. The thin mediastinal pleura and the poor tolerance to pneumonectomy explain the high mortality rate of our intrapleural chemotherapy setup (10/21). Nevertheless, all of the animals that underwent high-pressure intrapleural chemotherapy without lung resection survived the procedure. Another limitation is the lack of a model of pleural carcinomatosis in the pig, which could be used to evaluate the antitumor effect of high-pressure intrapleural chemotherapy. Models of malignant pleural effusion exist in rats, but rats have a single pleural space thus precluding high-pressure pleural infusion [15]. This experimental model is based on the similarities between the peritoneum and pleura, but the interest of high-pressure to increase the penetration of chemotherapy in pleura has to be proved is further studies, with tissue samples in the same way as in peritoneal studies [25].

Chemotherapy associating cisplatin and gemcitabine was well tolerated when diluted in 4 l of saline solution and maintained for 40 to 60 min in the right pleural cavity under a constant pressure of 20 cm H_2_0. In this study, we selected cisplatin and gemcitabine for their rapid cytotoxic effect against a variety of human cell lines originating from cancers that frequently affect the pleura. Moreover, the cisplatin/gemcitabine combination is synergistic on lung cancer and mesothelioma [26,27]. We used 30 mg/l (120 mg for 4 l) cisplatin and 100 mg/l (400 mg for 4 l) gemcitabine for intrapleural chemotherapy in 50-60 kg pigs. These total doses are in the range of those used for intravenous administration in patients (i.e. 120 mg cisplatin and 1500 mg gemcitabine for a 60-kg patient treated for lung cancer). The metabolism of gemcitabine to inactive dFdU was negligible in the pleura, but greater in the blood (on average 30%). As a consequence of this low systemic diffusion, no intra-operative changes were recorded in chemotherapy-treated animals as compared to those which received drug-free saline in the pleural space. This low systemic diffusion is also of interest with regard to the absence of potential toxicity. Animals did well during the operation and until the autopsy 1 month later, without neurological, haematological, or renal toxicity. The pleural adherences seen at the autopsy could be an advantage as they ensure solid pleurodesis and reduce the risk of recurrent pleural effusion. Five pigs were necessary for this tolerance study given the good peri-operative tolerance of the drug-free filling group (3 pigs for the 20 cmH_2_O level of pressure). Future studies to establish the pharmacokinetics of high-pressure intrapleural chemotherapy precisely (both in the blood and the tissue) at different levels of pressure will contribute to the next step of this research program.

## Conclusion

This feasibility study reports for the first time the concept of high-pressure intrapleural chemotherapy. In pigs, this technique using cisplatin and gemcitabine was well tolerated in the absence of pneumonectomy at 20 cmH_2_O. Given the similarity between the pleura and peritoneum, high-pressure could enhance the penetration of drugs with minimal systemic diffusion, but this needs to be assessed in further experiments.

## Competing interests

The authors declare that they have no competing interests.

## Authors' contributions

OF performed the experiments and drafted the manuscript. PBP performed the experiments. POD participated in the design of the study (in vivo) and performed the statistical analysis. GM participated in the experiments (haemodynamic parameters). BR performed the assay. SL participated in the design of the study (in vitro). BC and AB conceived the study, and participated in its design and coordination. All authors read and approved the final manuscript.
